# Community Partitioning over Feature-Rich Networks Using an Extended K-Means Method

**DOI:** 10.3390/e24050626

**Published:** 2022-04-29

**Authors:** Soroosh Shalileh, Boris Mirkin

**Affiliations:** 1 Center for Language and Brain, HSE University, Myasnitskaya Ulitsa 20, 101000 Moscow, Russia; 2 Department of Data Analysis and Artificial Intelligence, HSE University, Pokrovsky Boulevard, 11, 101000 Moscow, Russia; bmirkin@hse.ru or; 3 Department of Computer Science and Information Systems, Birkbeck University of London, Malet Street, London WC1E 7HX, UK

**Keywords:** node-attributed networks, feature-rich networks, community detection, cluster analysis, data recovery, K-means clustering, nonsummability assumption

## Abstract

This paper proposes a meaningful and effective extension of the celebrated K-means algorithm to detect communities in feature-rich networks, due to our assumption of non-summability mode. We least-squares approximate given matrices of inter-node links and feature values, leading to a straightforward extension of the conventional K-means clustering method as an alternating minimization strategy for the criterion. This works in a two-fold space, embracing both the network nodes and features. The metric used is a weighted sum of the squared Euclidean distances in the feature and network spaces. To tackle the so-called curse of dimensionality, we extend this to a version that uses the cosine distances between entities and centers. One more version of our method is based on the Manhattan distance metric. We conduct computational experiments to test our method and compare its performances with those by competing popular algorithms at synthetic and real-world datasets. The cosine-based version of the extended K-means typically wins at the high-dimension real-world datasets. In contrast, the Manhattan-based version wins at most synthetic datasets.

## 1. Introduction: The Problem and Our Approach

Community detection in networks is an activity oriented at various applications from sociology to biology to computer science. The corresponding data structure is network, or graph, of objects, called nodes, that are interconnected by pair-wise edges or links. Additionally, a feature-rich (or node-attributed) network is supplied with a set of features characterizing nodes [1,2,3].

We consider a community to be a set of relatively densely interconnected nodes which also are similar in the feature space. The problem is regarded as such: given a feature-rich network, find a partition of the node set such that each part is a community. Many approaches have been proposed for identifying communities in feature-rich networks; for recent reviews see, for example, [3,4,5,6,7,8].

In our view, they all can be classified into two categories: heuristics and data modelling. Unlike heuristic approaches, those in data modelling involve an important characteristic: evaluation of the degree of correspondence between the data and found solutions. In this category, one may distinguish between theory-driven and data-driven approaches. A theory-driven approach involves a model for data generation leading to a probabilistic distribution, parameters of which can be recovered from the data (generative modelling).

In contrast, data-driven approaches involve no models for data generation but rather focus on the dataset as is. The dataset in this set of approaches is considered as an array of numbers to be recovered by decoding a model that “encodes” the data. As the author in [9] described, some of the popular methods of data analysis such as K-means clustering and singular value decomposition based on principal component analysis naturally fall within this category of methods.

This paper belongs to the data-driven modeling approach. Our data-driven model conventionally assumes a hidden partition of the node-set in non-overlapping communities, supplied with hidden parameters encoding the average link values in the network space and central community points in the feature space. This model has been described in our earlier publications [10,11], in which we developed a set of double-greedy algorithms. These algorithms find clusters, a.k.a. communities, one-by-one, not simultaneously (a ‘greedy’ strategy) so that each community is built one-by-one also, so that at each step, only one node is to be added to it (another ‘greedy’ strategy). Although quite competitive in terms of cluster recovery, these computations are rather time consuming.

The goal of this paper is to propose a different algorithm to allow processing larger datasets. Specifically, the paper develops an analogue to the popular K-means algorithm to minimize the summary least-squares criterion for the models of both network link data and feature matrix. We should point out that such a development became possible only because we had introduced, in [11], what we call the nonsummability mode. According to this model, the network data matrix is represented by community central vectors $\lambda_{k}=\left(\lambda_{kj}\right)$ which are similar to those in the feature space. Therefore, every community obtains a two-fold center, one fold in the feature space, the other in the network space. Such a representation allows us to develop a version of K-means in a combined data space as an alternating minimization of the combined least-squares criterion.

The combined criterion’s dimension is the total number of network nodes and features, which may be rather high indeed. That is usually a realm of the so-called curse of dimensionality which leads us to develop two additional versions of the method using the cosine distance and the Manhattan distance supplementing the squared Euclidean one. The cosine distance has been experimentally proven to lead to superior results in recommender systems (see, for example, [12]). The Manhattan distance has been comprehensively studied in [13] for the Wi-Fi positioning algorithm, in which it appeared to be a proper distance metric in some cases.

We conduct a comprehensive set of experiments to test the performance of the newly proposed techniques. Our experiments show that this approach can indeed recover hidden clusters in feature-rich networks. Moreover, it is competitive against existing state-of-the-art algorithms.

A part of this work was presented at the 2021 Complex Networks and their Applications International Conference (30 November–2 December 2021) [14]. The current paper differs, first of all, by the extent of detail reported so that the paper length has increased five-fold. Second, the experimental part has been significantly expanded by adding three larger real-world datasets and by increasing the number of synthetic datasets by 50%. Third, one more version of the method has been added, that one using the Manhattan distance, in addition to versions based on the cosine and squared Euclidean distances, to further extend the experimental part.

The paper is structured as follows. Section 2 describes our proposed method. Section 3 describes the setting of our experiments for testing the algorithms. Section 4 and Section 5 present results of our experiments. We draw conclusions in Section 6.

### 1.1. Related Work

#### 1.1.1. General

A wide variety of approaches have been developed for community detection so far. A reviewing paper [4] proposes a nice system for distinguishing among them. It classifies all the work on community detection in feature-rich networks according to the stage at which the two data types, network and features, are merged together. This may occur before the process of community detection begins (early fusion), within the process (simultaneous fusion), and after the process (late fusion). While referring an interested reader to look at the references in [4], we would like to add some details over here. We note that the methods of our interest, i.e., model-based ones, naturally fall within the simultaneous fusion stage.

In contrast, all methods related to the early fusion stage must be heuristic. Among them, one should distinguish two streams of work related to the way of fusion. Indeed, the two data sources can be converted, before detection of communities begin, into either network-only format (this can be dubbed ‘network augmenting’) or features-only format (this can be dubbed ‘network converting’). In the next two subsections, a brief review of these approaches is given.

#### 1.1.2. Augmenting the Network Structure

Methods in this category score similarities between features of nodes using various metrics. Paper [15] uses a matching index similarity. Paper [16] considers three groups of methods: (1) clustering coefficient similarity, (2) common neighbor similarity, (3) node attribute similarity. Stochastic random walk methods are another popular tool in this area [17,18,19]. In [20] Kohonen’s self-organizing maps are applied, whereas [21] uses Pagerank [22] as the cornerstone.

In a nutshell, once the node-to-node similarities are determined, there are two ways for augmenting the graph structure: either by (a) assigning greater weights to edges between more similar nodes, or by (b) inserting additional nodes to create a more homogeneous graph structure. After this, any graph-structuring algorithm can be applied, in a modified form. Methods described in [8,21,23] are instances of this approach.

#### 1.1.3. Converting the Network Structure into Feature Space

In [24], authors at first use a spectral analysis technique to map the network data into a low-dimensional Euclidean space. Then, a DENsity-based CLUstEring algorithm (DENCLUE) [25] runs to detect communities in the network. Paper [26] formulates the community detection problem as a class separability problem, which allows the simultaneous handling of many classes of communities and a diverse set of structural properties. The separability of classes provides information on the extent to which different communities come from the same (or fundamentally different) distributions of feature values.

The so-called “signal diffusion process” converts the network structure into geometrical relations of vectors in a Euclidean space in [27]. After this conversion, a modified version of K-means algorithm applies to detect the communities. In [28], the authors propose a quasi-isometric mapping to transform network structure into a time series, so that the machinery of time-series can be applied, with a follow-up K-means clustering.

The so-called network embedding approaches (see [29,30]) fall within this category too.

#### 1.1.4. Model-Based Community Detection

Here, we are going to give some references to papers developing model-based methods for community detection in feature-rich networks.

In [31], a statistical joint clustering criterion is proposed. In [32], a discriminative semi-supervised method is proposed.

The theory-driven approach may involve both the maximum likelihood and Bayesian criteria for fitting a probabilistic models for data generation. Many methods in this category involve stochastic block models (SBM) [33,34]. Methods in [1,2,35] are based on Bayesian inferences. In [36], the authors propose a clustering criterion to statistically model interrelation between the network structure and node attributes.

As for data-driven modeling, we can indicate the following works here. There exist several versions of non-negative matrix factorization for the problem of community detection (see [37,38]). This category of methods approximates the data by using matrix factorization techniques. Papers [39,40,41] propose combined criteria and corresponding methods for finding suboptimal solutions. The criteria are based on the least-squares approach applied to some derived data rather than the original ones. In contrast, Ref. [42] summarizes the data as observed by using a different clustering criterion, the minimum description length (MDL). Therefore our papers [10,11] fill in the gap by developing least-squares methods oriented at recovery of the datasets as they are. This paper extends this approach by developing a less computing-intensive version.

## 2. Least-Squares Criterion and Extended K-Means

### 2.1. The Summability and Non-Summability Assumptions

To model a community in a feature-rich network, one usually represents it by its central ‘standard’ point in the feature space and a single aggregate intensity value over the entire network data.

Let us denote the community set to be found by $S=\{S_{1},S_{2},\ldots ,S_{K}\}$, and community $S_{k}$’s standard point by $c_{k}=\left(c_{kv}\right)$ (v∈V,k=1,…,K). We define our model in the feature space $Y=(y_{iv})$ (where i∈I are the network nodes and v∈V, features) as:

(1) $$y_{iv}=\sum_{k=1}^{K}c_{kv}s_{ik}+f_{iv},\;\;i\in I,v\in V.\;$$

where $f_{iv}$ are residuals which should be made as small as possible.

Let us denote the aggregate intensity weight of community $S_{k}$ (k=1,2,…K), by $\lambda_{k}$. Then the network link data matrix $P=(p_{ij})$ (i,j∈I) can be modeled as:

(2) $$p_{ij}=\sum_{k=1}^{K}\lambda_{k}s_{ik}s_{jk}+e_{ij},i,j\in I,$$

where $e_{ij}$ are residuals to be minimized.

Equations (1) and (2) express the data-recovery essence of our model as follows. The observed data, both feature values and network links, can be reconstructed from the structure—the partition *S* and its parameters $c_{k},\lambda_{k}$—to be found, up to relatively small residuals according to these equations. The data recovery (or data reconstruction) approach currently has become a linchpin in data science.

In [10], we applied a combined least squares criterion to Equations (1) and (2) complemented with an iterative extraction principle [43], to arrive at the so-called SEFNAC method, which stands for Sequentially Extracting Feature-rich Network Addition Clustering, for obtaining communities one-by-one.

At each step of the SEFNAC method, a single cluster represented by a binary 0/1 vector $s=(s_{i})$, i∈I, so that the cluster combines those *i* at which $s_{i}=1$, is sought to maximize criterion

(3) $$\begin{array}{c} G\left(s\right)=2\rho \sum_{i,v}y_{iv}c_{v}s_{i}-\rho \sum_{v}c_{v}^{2}\sum_{i}s_{i}^{2}\\ +2\xi \lambda \sum_{i,j}p_{ij}s_{i}s_{j}-\xi \lambda^{2}\sum_{i}s_{i}^{2}\sum_{j}s_{j}^{2} \end{array}$$

over those i,j∈I that do not belong to the previously extracted clusters. Values ρ and ξ are expert-driven constants to balance relative weights of the two sources of data, network links and feature values.

In SEFNAC, criterion (3) is locally maximized by adding nodes one-by-one. SEFNAC appears to be competitive in terms of cluster recovery. Moreover, it allows for deriving the number of clusters from the data rather than by pre-specifying beforehand, in contrast to other approaches. However, the SEFNAC algorithm is time-consuming and does not allow processing large datasets, even in spite of the fact that it involves two simplifying assumptions by admitting that both individual clusters and cluster partitions can be found in a greedy one-by-one manner.

In [11], the authors introduced two different modes for interpreting link data: (a) summability and (b) nonsummability. In the summability mode, all link scores are considered as measured in the same scale. The nonsummability mode relates to the case at which each node’s links are considered as scored in different scales. An example of nonsummability: consider two sets of internet sites—one to provide physics instruction, the other to sell goods. These sets obviously differ both in the numbers of visitors and time spent at them. The former is is greater at physics instruction, whereas the latter greater at selling goods sites.

The nonsummability mode requires modifying the network link partitioning model by introducing column-dependent intensity weights $\lambda_{kj}$ rather than just $\lambda_{k}$, so that the following equations should hold:

(4) $$p_{ij}=\sum_{k=1}^{K}\lambda_{kj}s_{ik}+e_{ij},i,j\in I.$$

As shown in our papers [11,41], the nonsummability assumption allows one to somewhat reduce the computational intensity of the double-greedy iterative extraction approach without decreasing the quality of cluster recovery, although not without some quirks, as explored by the authors in [41]. Moreover, the there-developed algorithms still allow for automatic derivation of the number of clusters. However, they cannot be consistently applied at large datasets with thousands nodes because of their limited computational capacity. In the next section, we are going to tackle this challenge.

### 2.2. The Criterion and Its Alternating Minimization

Let us apply the least-squares approach to Equations (1) and (4). A combined sum of the squared residuals can be expressed as:

(5) $$\begin{array}{c} F\left(s_{ik},c_{kv},\lambda_{kj}\right)=\rho \sum_{i,v}{\left(y_{iv}-\sum_{k=1}^{K}c_{kv}s_{ik}\right)}^{2}+\xi \sum_{i,j}{\left(p_{ij}-\sum_{k=1}^{K}\lambda_{kj}s_{ik}\right)}^{2}. \end{array}$$

where factors ρ and ξ balance the relative weights of the two sources of data, network links and feature values.

The goal is to recover a hidden membership matrix $S=(s_{ik})$, community centers in the feature data space $c_{k}=\left(c_{kv}\right)$, and community centers in the network data space $\lambda_{k}=\left(\lambda_{kj}\right)$, by minimizing criterion (5).

This can be reformulated by using matrix notation. Recall that *Y* is a N×V feature matrix and *P* is a N×N link matrix. Denote a K×V matrix of cluster centers in the feature space by $C=(c_{kv})$ and a K×n matrix of cluster centers in the space of network matrix, by $\Lambda =(\lambda_{kj})$. Let $S=(s_{ik})$ be a 1/0 matrix of node belongingness to clusters. Then the model expresses approximate matrix factorization equations Y=SC and P=SΛ, whereas the least-squares criterion in (5) is $F=\rho Tr\left[{\left(Y-SC\right)}^{T}\left(Y-SC\right)\right]+\xi Tr\left[{\left(P-S\Lambda \right)}^{T}\left(P-S\Lambda \right)\right]$, where Tr(A) is the trace, the sum of diagonal entries, of matrix *A*.

Optimizing criterion (5) is computationally expensive and cannot be solved exactly in reasonable time. We adopt the so-called alternating minimization strategy, which is exemplified by the batch K-means algorithm in feature spaces [44]. This approach can be applied here too, because the variables in the criterion (5) can be divided in two groups to be sought separately. These groups are: (i) partition $\left\{S_{k}\right\}$, or corresponding belongingness matrix $S=(s_{ik})$ in which $s_{ik}=1$ if $i\in S_{k}$ and $s_{ik}=0$, otherwise; (ii) standard points $c_{k}=\left(c_{kv}\right)$ and $\lambda_{k}=\left(\lambda_{kj}\right)$, k=1,2,…,K.

An alternating minimization method works in iterations consisting of two steps each: (1) given centers $c_{k}$, $\lambda_{k}$, find partition $\left\{S_{k}\right\}$ minimizing criterion (5) with respect to *S*; (2) given partition $\left\{S_{k}\right\}$, find centers $c_{k}$, $\lambda_{k}$ minimizing criterion (5) with respect to all possible $c_{k}$, $\lambda_{k}$, k=1,…K.

To run step (1), let us define distances between nodes and centers. In the feature space, node i∈I is expressed by vector $y_{i}=\left(y_{iv}\right)$, v=1,…,V, and in the network space, by *i*-th row of matrix *P*, $p_{i}=\left(p_{ij}\right)$, j=1,…,N. The squared Euclidean distances between node *i* and standard vectors $c_{k}=\left(c_{kv}\right)$ and $\lambda_{k}=\left(\lambda_{kj}\right)$ are defined as $d_{e}\left(y_{i},c_{k}\right)=\sum_{v}{\left(y_{iv}-c_{kv}\right)}^{2}$ and $d_{e}\left(p_{i},\lambda_{k}\right)=\sum_{j}{\left(p_{ij}-\lambda_{kj}\right)}^{2}$.

Substituting these expressions in the criterion (5) and recalling that, for any i∈I, $s_{ik}=1$ can be for one *k* only, we obtain

(6) $$F\left(S,c,\lambda \right)=\sum_{k=1}^{K}\sum_{i\in S_{k}}\left[\rho d_{e}\left(y_{i},c_{k}\right)+\xi d_{e}\left(p_{i},\lambda_{k}\right)\right]$$

To minimize (6), one needs to apply the following Minimum Distance rule for determining an optimal partition at given standard points $c_{k}$ and $\lambda_{k}$. For any i∈I, assign *i* to that cluster $S_{k}$ for which the combined distance $d_{e}\left(i,k\right)=\rho d_{e}\left(y_{i},c_{k}\right)+\xi d_{e}\left(p_{i},\lambda_{k}\right)$ is minimum.

The optimal standard points $c_{k}$ and $\lambda_{k}$ are to be computed at a given partition $\left\{S_{k}\right\}$ as the within-cluster means:

(7) $$c_{kv}=\frac{\sum_{i\in S_{k}}y_{iv}}{|S_{k}|},\;\lambda_{kj}=\frac{\sum_{i\in S_{k}}p_{ij}}{|S_{k}|}.$$

This is easily derived from the first-order optimality conditions for the criterion (5).

Now we may formulate an extension of K-means clustering algorithm:

**K-Means Extended to Feature-Rich Networks (KEFRiN)**

1. Data standardization: standardize the features and the network links (see Section 4.1).
2. Initialization:
  - Choose a number of clusters, K>1;
  - Initialize seed centers: $C={\left\{c_{k}\right\}}_{k=1}^{K}$, $\Lambda ={\left\{\lambda_{k}\right\}}_{k=1}^{K}$, as described below.
3. Cluster update: given *K* centers in the feature space and *K* centers in the network space, determine partition ${\left\{S_{k}^{\prime }\right\}}_{k=1}^{K}$ using the Minimum Distance rule above.
4. Stop-condition: Check whether $S_{k}^{{}^{\prime }}=S_{k}$ for all k=1,…,K. If yes, stop and output partition ${\left\{S_{k}\right\}}_{k=1}^{K}$, and centers ${\left\{c_{k}\right\}}_{k=1}^{K},{\left\{\lambda_{k}\right\}}_{k=1}^{K}$. Otherwise, change $S_{k}$ for $S_{k}^{{}^{\prime }}$ at every *k*.
5. Center update: Given clusters ${\left\{S_{k}\right\}}_{k=1}^{K}$, calculate within-cluster means according to (7); go to Step 3.

To initialise the algorithm, we use a version of K-Means++ algorithm from [45] combined with MaxMin method from [9]. Specifically, we generate initial seeds from the set of nodes as follows.

**Seed initialization**

1. Start. Randomly choose an index r∈I and specify $c_{1}=y_{r}$, *r*-th row of *Y*, and $\lambda_{1}=p_{r}$, *r*-th row of *P*.
2. General step.
  (a) Given a set of already defined seeds, $\left(c_{1},\lambda_{1}\right),\ldots ,\left(c_{k},\lambda_{k}\right)$, compute the sum of combined distances $f\left(i\right)=d_{e}\left(i,1\right)+d_{e}\left(i,2\right)+\ldots +d_{e}\left(i,k\right)$ for all remaining i∈I. (Recall that $d_{e}\left(i,k\right)=\rho d_{e}\left(y_{i},c_{k}\right)+\xi d_{e}\left(p_{i},\lambda_{k}\right)$ for all i∈I and k=1,…,K.)
  (b) Define the next, k+1-th center using that node *i* for which f(i) is maximum.
3. If k+1 is equal to *K*, halt. Otherwise, set k:=k+1 and go to the General step.

### 2.3. Using Manhattan Distance in KEFRiN

Given two vectors $f=(f_{t})$ and $g=(g_{t})$, t=1,…,T, the Manhattan distance between them is defined as

(8) $$d_{m}\left(f,g\right)=\sum_{t}\left|f_{t}-g_{t}\right|.$$

Using the Manhattan distance $d_{m}\left(.\right)$, the Equation (6) can be rewritten as $F_{n}\left(S,c,\lambda \right)=2\sum_{k=1}^{K}\sum_{i\in S_{k}}\left[\rho d_{m}\left(y_{i},c_{k}\right)+\xi d_{m}\left(p_{i},\lambda_{k}\right)\right]$. With this, we propose one more version of K-means extended to feature-rich networks: using the Manhattan metric $d_{m}$ as the distance in KEFRiN algorithm above.

### 2.4. Using Cosine Distance in KEFRiN

Given two vectors $f=(f_{t})$ and $g=(g_{t})$, t=1,…,T, the cosine between them is defined as

(9) $$cos\left(f,g\right)=\frac{<f,g>}{∥f∥∥g∥}=\frac{\sum_{t}f_{t}g_{t}}{\sqrt{\sum_{t}f_{t}^{2}}\sqrt{\sum_{t}g_{t}^{2}}}.$$

One may say that the cosine of two vectors is just the inner product of them after they had been normed. The cosine gives rise to what is referred to as the cosine distance between *f* and *g*, $d_{c}\left(f,g\right)=1-cos\left(f,g\right)$.

In fact, the cosine distance between *f* and *g* is a half squared Euclidean distance between *f* and *g* after they had been normed. Indeed, assuming that *f* and *g* are normed, ∥f∥=∥g∥=1, we have $d_{e}\left(f,g\right)=\sum_{t}{\left(f_{t}-g_{t}\right)}^{2}=\sum_{t}f_{t}^{2}+\sum_{t}g_{t}^{2}-2\sum_{t}f_{t}g_{t}={∥f∥}^{2}+{∥g∥}^{2}-2<f,g>=2-2cos\left(f,g\right)=2d_{c}\left(f,g\right)$.

Therefore, under the assumption that all vectors $y_{i}$, $c_{k}$, $p_{i}$, and $\lambda_{k}$ occurring in the Equation (6) are normed, that equation can be rewritten as $F_{n}\left(S,c,\lambda \right)=2\sum_{k=1}^{K}\sum_{i\in S_{k}}[\rho d_{c}\left(y_{i},c_{k}\right)$ + $\xi d_{c}\left(p_{i},\lambda_{k}\right)]$.

This leads us to propose one more version of K-means extended to feature-rich networks: that using the cosine metric $d_{c}$ as the distance in KEFRiN algorithm above.

To distinguish between the three versions, we use the abbreviation KEFRiNe based on the squared Euclidean metric, KEFRiNm for the Manhattan distance case, and finally, the abbreviation KEFRiNc for that based on the cosine distance.

It should be pointed out that the three versions are different indeed: the $d_{c}$ based version of the criterion holds only at normed vectors. KEFRiNc algorithm assumes that all the data should be preprocessed so that all vectors $y_{i}$ and $p_{i}$ are normed. Moreover, the step of center updating in KEFRiNc involves a supplementary operation: after the vectors of within-cluster means are found, they must be normed. The norming operation breaks the property that the centers are optimal according to the least-squares criterion: they are not any more, which may affect the algorithm’s convergence.

To further specify KEFRiN algorithms, we should choose the values of weights ρ and ξ balancing the two data sources, features and network. In this paper, we take them to be equal, say, to 1.

We publish our Python source code of the KEFRiN methods at https://github.com/Sorooshi/KEFRiN, accessed on 2 April 2021.

## 3. Defining Experimental Framework for Testing KEFRiN

In this section, we specify our experimental setting. This includes: (1) the competing algorithms; (2) the real-world and synthetic datasets to which the algorithms are evaluatated; (3) a set of criteria for evaluation of the results by an algorithm; (4) a set of data pre-processing techniques.

### 3.1. Algorithms under Comparison

We compare the performance of our proposed methods with four algorithms of the model-based approach, Communities from Edge Structure and Node Attributes (CESNA) [36], Structure and inference in annotated networks (SIAN) [35], Sequential Extraction of Attributed Network Addition Clusters (SEANAC) [46] and, Deep Modularity Networks (DMoN) [47]. Author-made codes of these algorithms are publicly available. We also tested the algorithm PAICAN (Partial Anomaly Identification and Clustering in Attributed Networks) from [1]. Unfortunately, the computational results obtained by this algorithm were always less than satisfactory; therefore, we excluded PAICAN from this paper.

In the remainder of this subsection, we briefly describe CESNA, SIAN, SEANAC and DMoN algorithms.

**CESNA [36] overview**: The authors define two generative models, one for the graph and the other for attributes, and combine them together. They use equation $P_{uv}$ = $1-exp(-\sum_{c=1}^{C}F_{uc}F_{vc})$ to model the probability of an edge between nodes *u* and *v*. Unknown function $F_{uc}$ represents the membership of node *u* to community *c*. Similar logistic models, with parameter matrices *W*, are defined for binary attributes at nodes. Then values of latent variables *F* and *W* are inferred by maximizing the likelihood of the observed data. An author-supplied code for CESNA algorithm can be found at [48].

**SIAN [35] overview**: Assume that each node belongs to a community with the probability depending on the feature values at the node. Edges between nodes are formed independently at random, according to a distribution involving the node degrees. The likelihood is maximized with the Expectation-Maximisation (EM) algorithm. An author-supplied code for SIAN algorithm can be found at [35].

**SEANAC [46] overview**: One cluster at a time strategy is pursued by minimization of a least-squares criterion similar to that in in Equation (5), but applied for finding just one cluster only. The authors derive a complementary optimality criterion, which is locally optimized by adding nodes to cluster one-by-one [46]. A SEANAC code is publicly available at [49].

**DMoN [47] overview**: The algorithm finds a soft cluster assignment matrix by using Graph convolutional networks (GCNs) involving the given attribute matrix in the first layer of the network. An objective function used is inspired by the Modularity criterion [47].

### 3.2. Datasets

We use both real-world and synthetic datasets. We describe them in the following subsections.

#### 3.2.1. Real World Datasets

Table 1 briefly summarizes the eight real-world data sets under consideration. All the features are categorical, since two of the algorithms under comparison, CESNA and SIAN, are applicable only at categorical features.

Let us describe them in turn.

**Consulting Organisational Social Network (COSN) dataset [50]**: The nodes in this network correspond to employees in a consulting company. The (asymmetric) edges are formed in accordance with their replies to this question: “Please indicate how often you have turned to this person for information or advice on work-related topics in the past three months”. The answers are coded by 0 (I Do Not Know This Person), 1 (Never), 2 (Seldom), 3 (Sometimes), 4 (Often), and 5 (Very Often). Either of these 6 numerals is the weight of the corresponding edge. The Region feature is considered as the ground truth. More detail can be found in our papers [11,41].

**Lawyers dataset [51]**: The Lawyers dataset comes from a network study of corporate law partnerships. It is available for download at [56]. There is a friendship network between lawyers in the study. More detail can be found in our papers [11,41].

The combination of Office location and Status is considered the ground truth.

**World-Trade dataset [52]**: The World-Trade dataset contains data on trade between 80 countries in 1994. The link weights represent total imports by row-countries from column-countries for the class of commodities designated as ‘miscellaneous manufactures of metal’ to represent high technology products or heavy manufacture.

The node attributes are Continent, Position in the Structural World System in 1980 qnd in 1994, GDP. The GDP categories are defined as follows: ‘Poor’: GDP USD 4406.9; ‘Mid-Range’: USD 4406.9 < GDP USD 21,574.5, and ‘Wealthy’: GDP USD 21,574.5.

The Structural World System Position in 1980, according to Smith and White [57], is considered as the ground truth. More detail can be found in our papers [11,41].

**Malaria data set [53]**: The nodes are amino acid sequences containing six highly variable regions (HVR) each. The edges are drawn between sequences with similar HVRs 6. In this data set, there are two nominal attributes of nodes: (1) Cys Labels derived from a highly variable region HVR6 sequence; and (2) Cys-PoLV labels derived from the sequences adjacent to regions HVR 5 and 6.

The Cys Labels is considered as the ground truth.

**Parliament dataset [1]**: The 451 nodes correspond to members of the French Parliament. An edge is drawn if the corresponding MPs have signed a bill together. The 108 features are the constituency of MPs and their political party, as the authors describe it. The latter is considered the ground truth.

**Cora dataset [54]**: This dataset is obtained by collecting 2708 scientific publications and classifying them in seven categories. The citation network has 5429 links. The feature set in this dataset is a dictionary to consist of 1433 unique words.

**SinaNet dataset [8]**: This dataset is a microblog user relationship network extracted from the Sina-microblog website, http://www.weibo.com, accessed on 14 August 2009. The authors at first selected 100 VIP Sina-microblog users from 10 significant forums including finance and economics, literature and arts, etc. Then they extracted followers/followings of these 100 users and their published micro-blogs. Using the depth-first search strategy, they extracted three layers of user relationships and obtained 8452 users, 147,653 user relationships, and 5.5 million micro-blogs in total. They merged all micro-blogs published by a user to characterize the user interests. After removing users published less than 5000 words, they arrived at 3490 users and 30,282 relationships. They derived user topic distribution in the 10 forums obtained by the LDA topic modeling–these are 10-dimensional numerical features to characterize user interests.

**Amazon photo dataset [55]**: This dataset is a subset of the Amazon co-purchase graph for its photo section. The nodes represent goods with edges between those frequently bought together; node features are bag-of-word reviews. The class labels are product categories.

#### 3.2.2. Generating Synthetic Data Sets

Our synthetic data generator coincides with that described in our paper [11]. Thus, we describe further on rules for generating (a) network, (b) categorical features, and (c) quantitative features by following the description in [11].

**Generating network**

First, the number of nodes, *N*, and the number of communities, *K*, are specified. Then the cardinalities of communities are defined randomly subject two constraints (a) no community should have less than a pre-specified number of nodes, so that probabilistic approaches are applicable (in our experiments, this is set to 30); (b) and the total number of nodes in all the communities sums to *N*. We consider two settings for *N*: (a) N=200, for a small-size network, and (b) N=1000, for a medium-size network.

Given the community sizes, we populate them with nodes, that are specified just by indices. Then we specify two probability values, *p* and *q*. Every within-community edge is drawn with the probability *p*, independently of other edges. Every between-community edge is drawn independently with the probability *q*.

**Generating quantitative features**

The *K* and cluster sizes are generated at the stage of network generation.

To model quantitative features, we apply the design proposed in [58]. Each cluster is generated from a Gaussian distribution whose covariance matrix is diagonal with diagonal values uniformly random in the range [0.05, 0.1], specifying the cluster’s spread. Each component of the cluster center is generated uniformly random from the range α[−1,+1], where α, a real between 0 and 1, controls the cluster intermix. The smaller the α, the greater the chance that points from a cluster fall within the spreads of other clusters.

In addition to cluster intermix, the possibility of presence of noise in data is also taken into account. Uniformly random noise features are generated within an interval defined by the maximum and minimum values. In this way, 50% of the original data are replicated with noise features.

**Generating categorical features**

The number of categories for each feature is randomly chosen from the set {2,3,…,L} where L=10 for small-size networks and L=15 for the medium-size networks. Then cluster centers are generated by randomly selecting feature categories for each cluster separately, with respect to the constraint that no two centers may coincide at more than 50% of features.

Given a center of *k*-th cluster, $c_{k}=\left(c_{kv}\right)$, $N_{k}$ entities of this cluster are generated as follows. Given a pre-specified threshold of intermix, ϵ between 0 and 1, for every pair (i,v), $i=1:N_{k}$; v=1:V, a uniformly random real value *r* between 0 and 1 is generated. If r>ϵ, the entry $x_{iv}$ is set to be equal to $c_{kv}$; otherwise, $x_{iv}$ is taken randomly from the set of categories specified for feature *v*. Consequently, all entities in *k*-th cluster coincide with its center, up to errors specified by ϵ. The smaller the ϵ, the less homogeneous is the generated cluster.

A feature-rich network combining categorical and quantitative features is generated with equal numbers of quantitative and categorical features.

### 3.3. The Adjusted Rand Index as an Evaluation Criterion

We use two popular metrics of similarity between partitions: (1) Adjusted Rand Index (*ARI*) [59], and (2) Normalised Mutual Information (NMI) [60] for evaluation and comparison of cluster recovery results. Since in our experiments, these two measures lead to similar conclusions, for the sake of convenience, we report only *ARI* values.

To define the Adjusted Rand Index, let us recall the concept of contingency table from statistics. Given two partitions, $S=\{S_{1},S_{2},\ldots ,S_{K}\}$ and $T=\{T_{1},T_{2},\ldots ,T_{L}\}$, let the former represent the ground truth, whereas the latter represents the found clusters. A contingency table is a two-way table whose rows correspond to parts $S_{k}$ (k=1,2,…,K) of *S*, and its columns, to parts $T_{l}$ (l=1,2,…,L) of *T*. The (k,l)-th entry is $n_{kl}=\left|S_{k}\cap T_{l}\right|$, the frequency of (k,l) co-occurrence. The so-called marginal row *a* and marginal column *b* are defined by $a_{k}=\sum_{l=1}^{L}n_{kl}=\left|S_{k}\right|$ and $b_{l}=\sum_{k=1}^{K}n_{kl}=\left|T_{l}\right|$.

The Adjusted Rand Index is defined as:

(10) ARI(S,T)=∑k,lnkl2−[∑kak2∑lbl2]/N212[∑kak2+∑lbl2]−[∑kak2∑lbl2]/N2]

The closer the value of *ARI* to unity, the better the match between the partitions; *ARI* = 1.0 if and only if S=T. If one of the partitions consists of just one part, the set *I* itself, then *ARI* = 0.

## 4. Computationally Testing KEFRiN Methods

### 4.1. Data Pre-Processing

The results of KEFRiN and SEANAC methods depend on data standardization.

For feature data, we consider two popular standardization methods: (R)Range standardization: each of the features is centered by subtraction of its mean from all its values, and then normalized by dividing over its range, the difference between its maximum and minimum by (Z) Z-scoring: each of the features is centered by subtraction of its mean from all its values, and then normalized by dividing over its standard deviation;

We apply the two following network standardization methods:

(M) Modularity: Given an N×N similarity matrix $P=(p_{ij})$, compute summary values $p_{i+}=\sum_{j=1}^{N}p_{ij}$, $p_{+j}=\sum_{i=1}^{N}p_{ij}$, $p_{++}=\sum_{i,j=1}^{N}p_{ij}$ and random interaction scores $r_{ij}=p_{i+}p_{+j}/p_{++}$. Clear link weights from random interactions by changing $p_{ij}$ for $p_{ij}-r_{ij}$.

(S) Scale shift: Compute the mean link score $\pi =\sum_{i,j=1}^{N}p_{ij}/N^{2}$; change all $p_{ij}$ for $p_{ij}-\pi$.

Based on a systematic experimental study of effects of various standardization options, we selected options: (Z) for feature data and (M) for network link data, as those leading to, generally, best results at KEFRiN algorithms. The only exception to this rule applies when categorical features are present: then option (S) rather than (M) is used for network link data.

### 4.2. Experimental Validation of KEFRiN Methods at Synthetic Feature-Rich Networks

#### 4.2.1. KEFRiN on Synthetic Networks with Quantitative Features

Table 2 shows performance of KEFRiN methods at the selected pre-processing options over small-size networks with quantitative features only.

At no noise cases, KEFRiNm and KEFRiNc win four settings each. It is noteworthy that, on average, KEFRiNc obtains better results. Specifically, at the worst combinations of p,q,α parameters, it wins the competition with the *ARI* values of about 0.75. In the presence of noise, KEFRiNe wins two settings. These patterns are extended at medium-sized networks with quantitative features only (for the sake of brevity, the results are omitted).

#### 4.2.2. KEFRiN at Synthetic Networks with Categorical Features

Table 3 shows KEFRiN’s performance over small-sized and medium-sized networks with categorical features (at the selected standardization techniques).

In both cases, the small-sized data and medium-sized data, KEFRiNm wins overall. A maximum value, 0.787, by KEFRiNe at small-sized data looks like just a random splash balanced by a low value, 0.279, at its counter-part at medium-sized data, due to the randomness of the data. Still, one cannot help but notice that the performances are somewhat worse at the medium-sized data.

#### 4.2.3. KEFRiN at Synthetic Networks Combining Quantitative and Categorical Features

Table 4 and Table 5 present the performance of KEFRiN methods, in respect, over small-size and medium-size networks at which both quantitative and categorical features are present.

In Table 4, KEFRiNm generally wins in both cases, with and without noise. Moreover, one can see that presence of the noise does not much affect the performance of KEFRiN algorithms at the mixed scale data case. It looks that addition of categorical features to quantitative features acts as similar to addition of quantitative noise features to them.

KEFRiNc outperforms the competitors in the case of medium-size networks with no noise. In the presence of noise, KEFRiNc remains the winner on average, although it slightly loses to KEFRiNe in five out of the eight settings.

Overall, one may conclude that KEFRiNm mostly wins over KEFRiNe and KEFRiNc at synthetic datasets, with one notable exception: KEFRiNc wins at the medium-size mixed-scale feature datasets.

## 5. Experimental Comparison of Selected Methods

In this section, we describe our experimental results at comparison of the proposed methods with those selected as competition. In the first subsection, we compare the methods with synthetic datasets. We consider here only categorical features generated for the network nodes because some competing algorithms work only at this restriction. The second subsection is devoted to comparison of the methods at the real world feature-rich networks.

### 5.1. Comparison of Methods over Synthetic Networks with Categorical Features

Table 6 and Table 7 compare the performance of the algorithms under consideration at synthetic networks with categorical features, those small-sized and medium-sized, respectively.

Two methods, SEANAC and CESNA, dominate the Table 6 at small-size networks. However, at the “difficult” settings for (p,q,ϵ) in the last two rows, our methods SEANAC, KEFRiNc and KEFRiNe show much better performances. SEANAC remains the only winner at medium-sized networks, see Table 7, whereas CESNA’s performance decisively declines at the settings of the last two rows. DMoN and SIAN show relatively poor performances, especially at the medium-size datasets at which the performance of SIAN is undermined by the convergence issues. KEFRiN methods, especially KEFRiNm, perform relatively well getting the second-best position at many settings. One should also notice their relatively low computational cost.

### 5.2. Comparison of the Algorithms over Real-World Feature-Rich Network Data

Those pre-processing methods that lead, on average, to the largest *ARI* values have been chosen for the least-squares methods at each of the datasets. They are presented in Table 8.

Computational results by the algorithms under consideration over the real-world datasets are presented in Table 9.

Two algorithms dominate the Table 9, KEFRiNc and DMoN. In contrast, results by CESNA and KEFRiNm are obviously inferior here. The SIAN algorithm, which generally under-performed, unexpectedly produced the best—and a rather good—result at the Parliament dataset.

It should be noted that our results for DMoN at the Cora and Amazon photo datasets somewhat differ from those in [47], probably because we use the entire Cora and Amazon photo datasets, whereas [47] used subsets of them.

### 5.3. Comparison of Methods over Computational Complexity

The complexity of k-means method has been extensively studied (see [61] for a review). It is known that a single iteration of the method is rather computationally effective, taking, on average, no more steps than of the order of the squared number of objects, which can be reduced, with simple heuristics, to the order of the number of objects. However, the number of iterations, in a worst-case scenario, can be really high: its lower bound is super-polynomial. This theory, however is not supported by real-life computations.

Specifically, we ran a series of computations with algorithms under consideration to compare their performances in time needed to compute. Table 10 reports time, in seconds, taken by each of them over synthetic networks with categorical features over the easiest and the most challenging settings. The reported times have been achieved at a desktop computer Intel(R) (Core(TM) i9-9900K CPU /@ 3.60 GHz, RAM: 64 GB, HD: 1 TB SSD) under Ubuntu 18.0 Operating System).

As one can see, both KEFRiNm and KEFRiNc work much faster than the competition. This fast performance may have two reasons: (1) K-Means ++ seeds initialization leads to faster convergence; (2) the non-summability models have been implemented in a vectorized form.

Among our four competitors under consideration, CESNA and SIAN, in respect, can be considered the fastest and slowest competitors.

## 6. Conclusions

In this paper, we abandon our double-greedy approach for community detection in feature-rich networks [11,41]. We apply a different strategy here by exploiting our Nonsummability assumption for the network link data [11]. This allows us to minimize the least-squares criterion by alternatingly applying it in either of two spaces, the feature space and the similarity data space. This KEFRiN method is a straightforward extension of the conventional batch K-means clustering method. We operate with three versions of the algorithm, KEFRiNe, KEFRiNm and KEFRiNc, based on the squared Euclidean distance, Manhattan distance and the cosine distance, respectively. It appears they are competitive against a set of recent algorithms from the literature, especially over the running time, although no universal clear-cut winner emerges in our experiments.

There are two features distinguishing KEFRiN algorithms from others: (i) they admit both categorical and quantitative features; (ii) they need data pre-processing, both shift of the origin and normalization. The latter may be considered both a shortcoming (unfriendliness to the user) and an advantage (the user may affect the results by using a meaningful data standardization).

Among the directions for future work we would like to mention the following: (a) adapting KEFRiN algorithms to larger datasets; (b) investigating the effects of balancing weights ρ and ξ and, moreover, automating the choice of them; (c) developing a probabilistic framework in which KEFRiN-like algorithms would serve as model-fitting procedures analogous to EM-algorithms for fitting mixture-of-distributions models; (d) extending KEFRiN algorithms to incremental and/or kernel-based options; (e) investigating the issue of determining the “right” number of clusters;

## Figures and Tables

**Table 1:** Real world datasets.

Name	Vertices	Links	Attributes	Number of Communities	Ground Truth	Ref.
COSN	46	552	16	2	Region	[50]
Lawyers	71	339	18	6	Derived out-of-office and status features	[51]
World Trade	80	1000	16	5	Structural world system in 1980 features	[52]
Malaria HVR6	307	6526	6	2	Cys Labels	[53]
Parliament	451	11,646	108	7	Political parties	[1]
Cora	2708	5276	1433	7	Computer Science research area	[54]
SinaNet	3490	30,282	10	10	Users of same forum	[8]
Amazon Photo	7650	71,831	745	8	Product categories	[55]

**Table 2:** KEFRiN’s Performance at small-size networks with quantitative features only: the average and standard deviation of *ARI* index over 10 different data sets. Both cases, with and without noise, are considered. The best results are bold-faced.

	No Noise			With Noise		
p,q,α	KEFRiNe	KEFRiNc	KEFRiNm	KEFRiNe	KEFRiNc	KEFRiNm
0.9, 0.3, 0.9	0.818(0.163)	0.920(0.132)	**0.951(0.102)**	0.847(0.185)	**0.971(0.086)**	0.926(0.115)
0.9, 0.3, 0.7	0.823(0.119)	0.904(0.117)	**0.925(0.117)**	**0.866(0.112)**	0.837(0.138)	0.862(0.143)
0.9, 0.6, 0.9	0.792(0.179)	0.737(0.124)	**0.866(0.157)**	0.765(0.092)	0.738(0.174)	**0.770(0.176)**
0.9, 0.6, 0.7	0.796(0.180)	**0.865(0.135)**	0.802(0.184)	0.800(0.180)	0.765(0.162)	**0.806(0.177)**
0.7, 0.3, 0.9	0.849(0.128)	**0.909(0.119)**	0.880(0.152)	0.786(0.178)	**0.915(0.125)**	0.845(0.133)
0.7, 0.3, 0.7	0.809(0.098)	0.803(0.132)	**0.935(0.100)**	0.831(0.142)	0.760(0.200)	**0.944(0.112)**
0.7, 0.6, 0.9	0.499(0.184)	**0.753(0.162)**	0.303(0.086)	**0.558(0.174)**	0.544(0.164)	0.316(0.150)
0.7, 0.6, 0.7	0.595(0.189)	**0.742(0.125)**	0.306(0.114)	0.462(0.129)	**0.534(0.100)**	0.279(0.141)
Average	0.748	0.830	0.831	0.740	0.758	0.718

**Table 3:** KEFRiN’s performance at small-sized and medium-sized synthetic networks with categorical features: The average and standard deviation of *ARI* index over 10 different data sets. The best results are highlighted in bold-face.

	Small			Medium		
p,q,α	KEFRiNe	KEFRiNc	KEFRiNm	KEFRiNe	KEFRiNc	KEFRiNm
0.9, 0.3, 0.9	0.855(0.145)	0.922(0.119)	**0.961(0.079)**	0.508(0.205)	0.724(0.097)	**0.863(0.089)**
0.9, 0.3, 0.7	0.795(0.149)	0.819(0.142)	**0.863(0.138)**	**0.777(0.129)**	0.742(0.182)	0.762(0.184)
0.9, 0.6, 0.9	**0.787(0.149)**	0.726(0.097)	0.893(0.147)	0.279(0.204)	**0.652(0.110)**	0.894(0.074)
0.9, 0.6, 0.7	0.588(0.173)	0.711(0.145)	**0.821(0.120)**	0.766(0.180)	0.733(0.083)	**0.819(0.053)**
0.7, 0.3, 0.9	0.827(0.141)	0.877(0.130)	**0.951(0.099)**	0.364(0.247)	0.641(0.111)	**0.791(0.119)**
0.7, 0.3, 0.7	0.794(0.144)	0.795(0.117)	**0.877(0.137)**	**0.829(0.085)**	0.797(0.088)	0.759(0.092)
0.7, 0.6, 0.9	0.399(0.094)	0.819(0.142)	**0.865(0.119)**	0.426(0.246)	0.591(0.094)	**0.859(0.083)**
0.7, 0.6, 0.7	0.074(0.047)	**0.834(0.132)**	0.392(0.121)	0.671(0.196)	**0.773(0.070)**	0.695(0.074)
Average	0.640	0.812	0.828	0.578	0.710	0.810

**Table 4:** The average and standard deviation of *ARI* index over 10 different data sets for KEFRiN results at small-size networks combining quantitative and categorical features, with and without noise. The best results are highlighted in bold-face.

	No Noise			With Noise		
p,q,α	KEFRiNe	KEFRiNc	KEFRiNm	KEFRiNe	KEFRiNc	KEFRiNm
0.9, 0.3, 0.9	0.823(0.125)	0.752(0.096)	**0.869(0.132)**	**0.862(0.140)**	0.810(0.153)	0.859(0.146)
0.9, 0.3, 0.7	0.840(0.133)	0.769(0.101)	**0.944(0.114)**	0.864(0.137)	0.858(0.143)	**0.873(0.130)**
0.9, 0.6, 0.9	0.756(0.171)	0.809(0.138)	**0.817(0.179)**	0.733(0.184)	0.717(0.130)	**0.923(0.106)**
0.9, 0.6, 0.7	**0.831(0.185)**	0.716(0.122)	0.754(0.193)	0.708(0.223)	0.549(0.186)	**0.845(0.150)**
0.7, 0.3, 0.9	0.872(0.129)	0.750(0.078)	**0.897(0.129)**	0.713(0.185)	**0.881(0.109)**	0.851(0.152)
0.7, 0.3, 0.7	0.782(0.155)	0.681(0.078)	**0.848(0.152)**	0.840(0.130)	0.647(0.143)	**0.899(0.125)**
0.7, 0.6, 0.9	0.583(0.143)	**0.704(0.139)**	0.389(0.122)	**0.576(0.104)**	0.520(0.118)	0.244(0.130)
0.7, 0.6, 0.7	0.473(0.095)	**0.540(0.135)**	0.207(0.098)	0.370(0.123)	**0.421(0.106)**	0.183(0.059)
Average	0.745	0.715	0.716	0.708	0.675	0.710

**Table 5:** The average and standard deviation of *ARI* index over 10 different data sets for KEFRiN results at medium-size networks combining quantitative and categorical features, with and without noise. The best results are highlighted in bold-face.

	No Noise			With Noise		
p,q,α	KEFRiNe	KEFRiNc	KEFRiNm	KEFRiNe	KEFRiNc	KEFRiNm
0.9, 0.3, 0.9	0.570(0.121)	**0.834(0.044)**	0.697(0.122)	0.541(0.122)	**0.790(0.102)**	0.777(0.116)
0.9, 0.3, 0.7	0.540(0.158)	**0.801(0.051)**	0.686(0.124)	**0.784(0.110)**	0.733(0.103)	0.768(0.131)
0.9, 0.6, 0.9	0.641(0.068)	**0.747(0.071)**	0.601(0.075)	**0.699(0.085)**	0.645(0.061)	0.641(0.069)
0.9, 0.6, 0.7	0.672(0.082)	**0.722(0.059)**	0.573(0.061)	**0.655(0.091)**	0.617(0.046)	0.624(0.074)
0.7, 0.3, 0.9	0.614(0.085)	**0.853(0.048)**	0.578(0.134)	0.556(0.113)	**0.739(0.117)**	0.551(0.104)
0.7, 0.3, 0.7	0.543(0.081)	**0.773(0.060)**	0.574(0.113)	0.753(0.084)	**0.708(0.078)**	0.658(0.108)
0.7, 0.6, 0.9	0.385(0.120)	**0.726(0.058)**	0.180(0.139)	**0.640(0.106)**	0.593(0.135)	0.077(0.105)
0.7, 0.6, 0.7	0.255(0.050)	**0.608(0.037)**	0.102(0.079)	**0.512(0.057)**	0.483(0.021)	0.035(0.010)
Average	0.528	0.758	0.499	0.642	0.664	0.516

**Table 6:** Comparison of CESNA, SIAN, DMoN, SEANAC and KEFRiN algorithms on small-size synthetic networks with categorical features: The average and standard deviation of *ARI* index over 10 different data sets. The best results are shown in bold-face and the second-best ones are underlined.

Dataset	CESNA	SIAN	DMoN	SEANAC	KEFRiNe	KEFRiNc	KEFRiNm
0.9, 0.3, 0.9	**1.00(0.00)**	0.554(0.285)	0.709(0.101)	0.994(0.008)	0.886(0.116)	0.922(0.119)	0.895(0.173)
0.9, 0.3, 0.7	0.948(0.105)	0.479(0.289)	0.380(0.107)	**0.974(0.024)**	0.835(0.138)	0.819(0.142)	0.891(0.135)
0.9, 0.6, 0.9	0.934(0.075)	0.320(0.255)	0.412(0.109)	**0.965(0.013)**	0.963(0.072)	0.726(0.097)	0.868(0.202)
0.9, 0.6, 0.7	**0.902(0.063)**	0.110(0.138)	0.213(0.051)	0.750(0.117)	0.694(0.096)	0.711(0.145)	0.791(0.191)
0.7, 0.3, 0.9	0.965(0.078)	0.553(0.157)	0.566(0.105)	**0.975(0.018)**	0.788(0.117)	0.877(0.130)	0.937(0.124)
0.7, 0.3, 0.7	**0.890(0.138)**	0.508(0.211)	0.292(0.077)	0.870(0.067)	0.836(0.115)	0.795(0.117)	0.824(0.191)
0.7, 0.6, 0.9	0.506(0.101)	0.047(0.087)	0.345(0.064)	**0.896(0.067)**	0.762(0.169)	0.834(0.132)	0.379(0.174)
0.7, 0.6, 0.7	0.202(0.081)	0.030(0.040)	0.115(0.058)	**0.605(0.091)**	0.574(0.142)	0.540(0.107)	0.184(0.098)

**Table 7:** Comparison of CESNA, SIAN, DMoN, SEANAC, and KEFRiN algorithms over medium-size synthetic networks with categorical features; average and standard deviation of *ARI* index over 10 different datasets. The best results are shown in bold-face and second ones are underlined.

Dataset	CESNA	SIAN	DMoN	SEANAC	KEFRiNe	KEFRiNc	KEFRiNm
0.9, 0.3, 0.9	0.894(0.053)	0.000(0.000)	0.512(0.137)	**1.000(0.000)**	0.508(0.205)	0.724(0.097)	0.863(0.089)
0.9, 0.3, 0.7	0.849(0.076)	0.000(0.000)	0.272(0.073)	**0.996(0.005)**	0.777(0.129)	0.742(0.182)	0.762(0.184)
0.9, 0.6, 0.9	0.632(0.058)	0.000(0.000)	0.370(0.063)	**0.998(0.002)**	0.279(0.204)	0.652(0.110)	0.894(0.074)
0.9, 0.6, 0.7	0.474(0.089)	0.000(0.000)	0.168(0.030)	**0.959(0.032)**	0.766(0.180)	0.733(0.083)	0.819(0.053)
0.7, 0.3, 0.9	0.764(0.068)	0.026(0.077)	0.446(0.099)	**1.000(0.001)**	0.364(0.247)	0.641(0.111)	0.791(0.119)
0.7, 0.3, 0.7	0.715(0.128)	0.000(0.000)	0.228(0.077)	**0.993(0.002)**	0.829(0.085)	0.797(0.088)	0.759(0.092)
0.7, 0.6, 0.9	0.060(0.024)	0.000(0.000)	0.332(0.051)	**0.998(0.001)**	0.426(0.246)	0.591(0.094)	0.859(0.083)
0.7, 0.6, 0.7	0.016(0.008)	0.000(0.000)	0.133(0.016)	**0.909(0.035)**	0.671(0.196)	0.773(0.070)	0.695(0.074)

**Table 8:** The selected standardization options for the least-squares community detection methods at the real world datasets. Symbols R, Z, S, M, N stand for Range standardization, Z-scoring, Scale shift, Modularity and No Pre-processing, respectively.

Dataset	SEANAC		KEFRiNe		KEFRiNc		KEFRiNm	
	Y	P	Y	P	Y	P	Y	P
Malaria HVR6	Z	U	R	R	N	N	Z	M
Lawyers	R	S	Z	N	Z	N	Z	M
World Trade	R	R	N	N	Z	M	R	M
Parliament	Z	M	N	N	Z	N	R	M
COSN	Z	N	Z	N	Z	N	R	M
Cora	Z	M	N	N	N	N	Z	M
SinaNet	Z	M	Z	Z	Z	N	Z	M
Amazon Photo	N|A		Z	S	N	N	Z	M

**Table 9:** Comparison of CESNA, SIAN, DMoN, SEANAC, KEFRiNe and KEFRiNc algorithms with Real-world data sets; average values of *ARI* are presented over 10 random initializations. The best results are highlighted in bold-face; those second-best are underlined.

Dataset	CESNA	SIAN	DMoN	SEANAC	KEFRiNe	KEFRiNc	KEFRiNm
HRV6	0.20(0.00)	0.39(0.29)	0.64(0.00)	0.49(0.11)	0.34(0.02)	**0.69(0.38)**	−0.056(0.004)
Lawyers	0.28(0.00)	0.59(0.04)	**0.60(0.04)**	**0.60(0.09)**	0.43(0.13)	0.44(0.14)	0.415(0.085)
World Trade	0.13(0.00)	0.10(0.01))	0.13(0.02)	0.29(0.10)	0.27(0.17)	**0.40(0.11)**	0.048(0.013)
Parliament	0.25(0.00)	**0.79(0.12)**	0.48(0.02)	0.28(0.01)	0.15(0.09)	0.41(0.05)	−0.035(0.001)
COSN	0.44(0.00)	0.75(0.00)	0.91(0.00)	0.72(0.02)	0.65(0.18)	**1.00(0.00)**	0.493(0.056)
Cora	0.14(0.00)	0.17(0.03)	**0.37(0.04)**	0.00(0.00)	0.00(0.00)	0.21(0.01)	−0.000(0.000)
SinaNet	0.09(0.00)	0.17(0.02)	0.28(0.01)	0.21(0.03)	0.31(0.02)	**0.34(0.02)**	0.001(0.000)
Amazon Photo	0.19(0.000)	N|A	**0.44(0.04)**	N|A	0.06(0.01)	0.43(0.06)	0.030(0.001)

**Table 10:** The execution time of methods under consideration at medium-size synthetic networks with categorical features at the nodes. The average of 10 different data sets at the same setting is reported in second. The fastest algorithm is shown in bold-face.

p,q,α	CESNA	SIAN	DMoN	SEANAC	KEFRiNe	KEFRiNc	KEFRiNm
0.9, 0.3, 0.9	38.265	856.785	124.698	492.006	2.434	2.389	**0.1946**
0.7, 0.6, 0.7	83.961	2674.541	207.541	476.251	2.859	3.131	**0.2261**

## Data Availability

https://github.com/Sorooshi/KEFRiN (accessed on 26 April 2022).
